# Dynamic electrocatalyst with current-driven oxyhydroxide shell for rechargeable zinc-air battery

**DOI:** 10.1038/s41467-020-15853-1

**Published:** 2020-04-23

**Authors:** Ya-Ping Deng, Yi Jiang, Ruilin Liang, Shao-Jian Zhang, Dan Luo, Yongfeng Hu, Xin Wang, Jun-Tao Li, Aiping Yu, Zhongwei Chen

**Affiliations:** 1 Department of Chemical Engineering, Waterloo Institute for Nanotechnology, Waterloo Institute for Sustainable Energy, University of Waterloo, Waterloo, ON N2L 3G1 Canada; 2 College of Energy, Xiamen University, Xiamen, 361005 People’s Republic of China; 3 Canadian Light Source, University of Saskatchewan, Saskatchewan, SK S7N 0X4 Canada; 4 South China Academy of Advanced Optoelectronics and International Academy of Optoelectronics at Zhaoqing, South China Normal University, Guangdong, 510631 People’s Republic of China

**Keywords:** Batteries, Electrocatalysis

## Abstract

Recent fruitful studies on rechargeable zinc-air battery have led to emergence of various bifunctional oxygen electrocatalysts, especially metal-based materials. However, their electrocatalytic configuration and evolution pathway during battery operation are rarely spotlighted. Herein, to depict the underlying behaviors, a concept named dynamic electrocatalyst is proposed. By selecting a bimetal nitride as representation, a current-driven “shell-bulk” configuration is visualized via time-resolved X-ray and electron spectroscopy analyses. A dynamic picture sketching the generation and maturation of nanoscale oxyhydroxide shell is presented, and periodic valence swings of performance-dominant element are observed. Upon maturation, zinc-air battery experiences a near two-fold enlargement in power density to 234 mW cm^−2^, a gradual narrowing of voltage gap to 0.85 V at 30 mA cm^−2^, followed by stable cycling for hundreds of hours. The revealed configuration can serve as the basis to construct future blueprints for metal-based electrocatalysts, and push zinc-air battery toward practical application.

## Abstract (web summary)

Interest in rechargeable Zn-air batteries has been renewed in recent years, however, their oxygen electrocatalysts remain not fully understood. Here the authors reveal the presence of a current-driven oxyhydroxide shell in a so-called dynamic eletrocatalyst that enables optimized battery performance.

## Introduction

Zinc–air (Zn–air) battery is widely recognized as one of the next-generation sustainable electrochemical energy-storage systems because of its high energy density, economic, and safety merits^1–3^. Previously, limitations in the availability and rechargeability of oxygen electrocatalysts have hindered their popularization, but blooming efforts on exploring suitable and durable candidates to catalyze cathode reactions have led to the recent rejuvenation of this century-old technology^4–6^. As such, huge families of materials have been investigated, including metals, alloys, oxides, sulfides, nitrides, phosphides, and their derived composites with carbon^2,7–18^. To this day, exploration for ideal bifunctional electrocatalysts remains to be the research mainstream, but attention is shifting toward understanding the relationship between battery performance and physiochemical properties of electrocatalysts for performance breakthrough. In particular, a debate over the actual active phases during cycling of these electrocatalysts is raised recently, and conflicting results have perplexed the research community^11,19,20^. Increasing reports on spectroelectrochemistry toward the half-reactions, that is, oxygen reduction (ORR) or evolution reactions (OER), in three-electrode systems suggest that an unexplored chasm is at work. For instance, Hu and coworkers reported both nickel iron diselenide and nickel phosphide as efficient and long-lasting OER electrocatalysts, but neither of them is static stereotype under oxidation voltage in alkaline electrolyte^21,22^. This contradiction was resolved with observations of surficial phase transformation from these compounds to their derived oxides or oxyhydroxides, which also aligns with the results on metal oxides, sulfides, and nitrides^19,23–25^. Furthermore, the latest attention has been drawn toward ORR electrocatalysts, in which an alteration was also demonstrated^26^.

These studies expose an often-overlooked fact that directly assuming the native state of metal-based electrocatalyst as the active representation in Zn–air battery operation may lead to false correlation between material characteristics and performance, albeit the undeniable role of their native properties. In particular, the electrocatalyst evolution during cycling is often ignored, and limited efforts have been placed to identify the actual configuration of the electrocatalyst in the mid-way or post-cycling status. Therefore, a systematic study is in high demand to pry into this “black box” and reveal the underlying mechanism governing the current-driven transformation of the electrocatalyst. Two key factors are worth pointing out, one is that the potentiodynamic-driven methodology applied in ORR or OER measurements is different from the continuous galvanostatic technique for battery operation, and the other is that alkaline electrolyte used in Zn–air batteries is far more concentrated, so that a lower electrochemical barrier is required for valence variation of the principle elements. The different environment may cause variances in electrochemical behaviors in comparison with the standard three-electrode system. To be specific, it is predicted herein that a current-driven oxyhydroxide derived from the native materials will be generated and combined with its electrochemically unavailable bulk as a “shell-bulk” configuration; the performance-dominant elements within the shell will experience periodic swing in their chemical states during cycling.

Here, for a vivid description of the predicted evolution, a concept of a dynamic electrocatalyst is proposed for a rechargeable Zn–air battery. A time coordinate is added as the fourth axis onto the basis of conventional three-dimensional spatial space, and hence a dynamic picture is established following battery operation. As a proof of this concept, a bimetal nitride, (Co,Fe)_3_N, is selected as a typical representation for metal-based materials with two major considerations. First, based on both experimental and theoretical evidences, metal nitride intrinsically exhibits metallic nature with high electrical conductivity^17,19,27,28^. By further incorporating thin nitrogen-doped carbon coating, dual-metal, and morphological benefits, the electrocatalyst is capable of delivering a power density of 133 mW cm^−1^ on the start-up step and a discharge–charge voltage gap of 1.08 V at 30 mA cm^−2^. More importantly, its oxygen-free bulk can furnish an ideal arena to record the interfacial formation of the predicted oxygen-containing shell during cycling. By collecting the time-varying information at both macro- and microscales, it is unveiled that a hexagonal oxyhydroxide shell is generated, followed by gradual maturation of a shell-bulk configuration as the actual state in Zn–air battery operation. Thereafter, current-driven valence swings of surficial Co are observed, while Fe at the surface and the bulk regions remains relatively inert. As a result, it is reflected electrochemically by an increase in power density up to 234 mW cm^−2^, shrink of voltage gap to 0.85 V at 30 mA cm^–2^, and long-lasting stability for over 300 h in Zn–air battery.

## Results

### Native properties

**Fig. 1:**
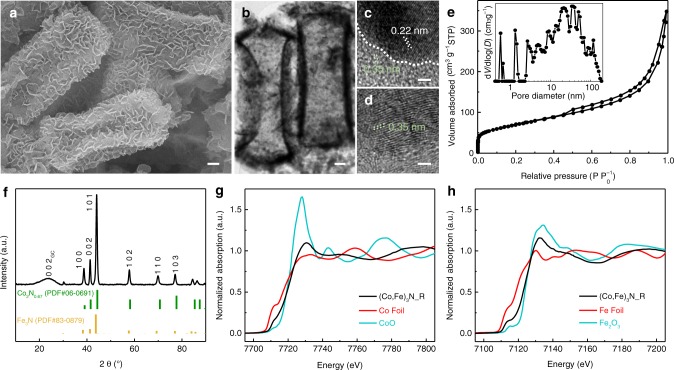
Morphology and native structural state. **a** SEM and **b** TEM images of secondary nanocuboids. **c**, **d** HRTEM images at different regions. Scale bar: **a**, **b** 200 nm; **c**, **d** 2 nm. **e** N_2_ adsorption–desorption isotherms and the corresponding pore-size distribution. **f** XRD pattern of (Co,Fe)_3_N_R with the Co_2_N_0.67_ (PDF#06-0691) and Fe_3_N (PDF#83-0879) as the reference patterns. **g** Co and **h** Fe K-edge XANES spectra with the respective metal foils and oxides as the reference spectra.

A bimetal-layered double hydroxide was first obtained via a cation-carving method using Fe^II^ as a graving agent to attack Co-based nanocuboid precursor (Supplementary Fig. 1a). This method allows a series of ligand-release and re-coordination processes to obtain a secondary hollow structure with tunable Co/Fe ratio controlled by stoichiometric factor of Fe^II^ source. After dopamine polarization and subsequent ammonolysis, the target bimetal nitride is obtained with an elemental Co/Fe ratio of 5:4 according to the energy-dispersive spectroscopy (EDS, Supplementary Fig. 2). The product takes on a nanoplate-aggregated hollow nanocuboid morphology with uniform elemental distribution (Fig. 1a, b and Supplementary Fig. 1). Its N_2_ adsorption/desorption isotherms in Fig. 1e reveal a typical type-IV curve with a surface area of 251.3 m^2^ g^−1^ and hierarchical porosity that includes micro-, meso-, and macropores as indicated in the inset of Fig. 1e. Specifically, the micro- and mesopores originate from holes on primary nanosheets and gaps in-between (inset of Supplementary Fig. 1c), while the macropores are mainly attributed to the void cavity of the secondary nanocuboids.

X-ray diffraction (XRD, Fig. 1f) of products suggests a typical hexagonal crystal structure indexed well to the reference patterns of Co_2_N_0.67_ (PDF#06-0691) and Fe_3_N (PDF#83-0879). By combining with the EDS result, the chemical formula of the bimetal nitride is confirmed to be (Co_0.56_Fe_0.44_)_3_N and marked as (Co,Fe)_3_N_R. High-resolution transmission electron microscopy (HRTEM) image in Fig. 1c demonstrates that the particle possesses a uniform crystal phase with lattice spacing of 0.22 nm belonging to the (0 0 0 2) facet. A carbon outer layer on a particle is also identified with a spacing of 0.35 nm and inconsistent N dopants (Fig. 1c, d). The clear boundary in-between verifies the critical role of a thin carbon layer in improving the air stability of nitride and protecting it from gradual oxidation in air^29,30^. Raman spectroscopy analysis of the sample confirms both graphitic and defective carbon characteristics (Supplementary Fig. 3)^31,32^. The thin carbon coating is not only beneficial for electrical conductivity and electrochemical performance, but also aids in maintaining the structural stability.

The electronic structure of (Co,Fe)_3_N_R is unveiled by Co and Fe K-edge X-ray adsorption near-edge structure (XANES) spectra. As demonstrated in Fig. 1g, Co K-edge curves of Co foil and (Co,Fe)_3_N_R both present a pre-edge hump at 7712 eV attributed to electron transitions from 1*s* to 3*d* as their metallic feature, which is almost absent in CoO because of the dipole-forbidden transition of octahedral Co^33^. In comparison with Co foil, (Co,Fe)_3_N_R presents a slightly weakened pre-edge hump as the nitride feature and an enlarged white-line crest, suggesting higher electron occupancies at the Co *3d* orbits and lower electron allocation at the *4p* orbits. This observation should be ascribed to its weaker 4*p*–3*d* hybridization at Co sites and the altered net charge distribution^34^. Similar conclusions can be made with the Fe K-edge spectrum with similar pre-edge characters (Fig. 1h)^35^. In addition, the inflection points of Co and Fe K edges of (Co,Fe)_3_N_R are both located close to their respective oxide references, suggesting similar electron offset toward N^36^.

### Electrochemical behaviors

Viability in battery application of the metal nitride was evaluated through a layer-by-layer Zn–air battery prototype using gas diffusion layer (GDL) sprayed with electrocatalyst as air electrode^37^. A performance reference with equal loading of commercial Pt/C and RuO_2_ on GDL was also prepared and tested in the same manner. Their polarization plots are shown in Supplementary Fig. 4. With a comparable open-circle voltage, (Co,Fe)_3_N_R first performs feebly at low current discharge. However, after a short period of expansion, its performance gap against the noble-metal reference begins to narrow at ~75 mA cm^−2^; its power density eventually peaks at 133 mW cm^−2^, which is superior to 113 mW cm^−2^ achieved by Pt/C + RuO_2_. As for the initial charging process of (Co,Fe)_3_N_R, it can be divided into two successive regions containing two intersection sites with Pt/C + RuO_2_ at 60 and 155 mA cm^–2^. Its initial rapid voltage rise suggests the inferior intrinsic activity, but it also demonstrates a much flatter slope compared with Pt/C + RuO_2_ after the initial surge. A step offset then occurs between the second intersection and the last period, and thereafter its uptrend is slightly accelerated to a comparable level to Pt/C + RuO_2_. All of the above phenomena reflected by polarization analysis point toward the electrochemical instability of the (Co,Fe)_3_N_R and its possible transformation with accumulated influence of galvanodynamic scans.

**Fig. 2:**
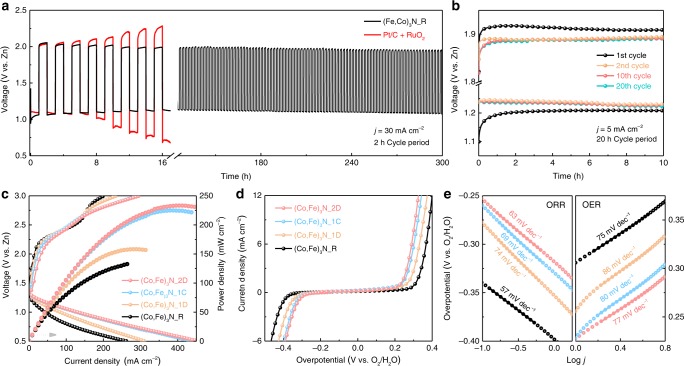
Electrochemical behaviors. **a** Cyclic performance of Zn–air batteries with the respective (Co,Fe)_3_N_R or an equal weight ratio mixture of 20% Pt/C and RuO_2_ in air cathode. Each cycle contains 1-h discharging and 1-h charging at a current density of 30 mA cm^–2^. **b** The comparison of discharge–charge profiles at different cycles with a current density of 5 mA cm^–2^ and a cycle period of 20 h. **c** The polarization curves and power density plots of Zn–air batteries processed to different electrochemical stages. **d** Bifunctional oxygen electrocatalytic activities of those air electrodes according to three-electrode system tests in O_2_ saturated with 0.1 M KOH electrolyte, and **e** the corresponding ORR or OER Tafel plots.

The galvanostatic discharge–charge profile of Zn–air battery using (Co,Fe)_3_N_R on GDL as air electrode is obtained under a current density of 30 mA cm^−2^ and 2-h cycling period (Fig. 2a). In comparison with the regular recession of Pt/C + RuO_2_, a slow activation process is observed for (Co,Fe)_3_N_R in the first several hours. In detail, during the initial discharge, (Co,Fe)_3_N_R shows a low start at 0.95 V (vs. Zn) against 1.10 V of Pt/C + RuO_2_, but it soon elevates with reducing their discrepancy to 0.03 V by the end. When turning to the initial charge, a smooth diminishment of 0.02 V is also observed in their voltage discrepancy. In the following cycles, the discharge–charge voltage gap of (Co,Fe)_3_N_R further narrows and eventually reaches a steady state of 0.85 V by the eighth cycle (16 h), while the gap expands rapidly to over 1.5 V for Pt/C + RuO_2_. This observation should be interpreted as an evolution of the (Co,Fe)_3_N_R that initiates at the first discharge, followed by a stepwise maturation in several hours. Afterward, the (Co,Fe)_3_N_R battery maintains a life cycle for over 300 h with a neglectable decay in discharge–charge voltage gap.

To magnify the maturation process of (Co,Fe)_3_N_R, another cycling experiment with a discharge–charge period of 20 h is conducted at 5 mA cm^−2^ (Fig. 2b and Supplementary Fig. 5). Similar phenomena are observed with a battery experiencing an iconic leap in its discharge plateau from 1.10 to 1.21 V after about 2 h, whereas its initial charge platform endures a smooth optimization from 1.92 to 1.91 V. In the subsequent cycle, a 0.04-V gain is observed to raise the discharge voltage to 1.25 V and a stable charge voltage of 1.89 V is achieved. Then, these battery parameters remain relatively constant for the following 700 h. Taking a panoramic view on the cycling behaviors, the maturation pathway of (Co,Fe)_3_N_R during battery operation can be divided into four clear stages along the time coordinate as indicated in Supplementary Fig. 5. They include the raw (Co,Fe)_3_N_R, the first discharged (Co,Fe)_3_N_1D, the first charged (Co,Fe)_3_N_1C, and the second discharged (Co,Fe)_3_N_2D. Besides the native properties of (Co,Fe)_3_N_R, (Co,Fe)_3_N_1D reflects latent generation of some new species, (Co,Fe)_3_N_1C enables the consolidation of the active phases, and finally (Co,Fe)_3_N_2D represents the matured configuration that realizes long-term cycling. Hereafter, their differences and influences on battery performance will be discussed in detail.

### Maturation pathway

The polarization curves of electrocatalysts processed to different electrochemical states are displayed in Fig. 2c. As shown, when comparing with the raw state, a higher power density of 158 mW cm^−2^ is delivered by (Co,Fe)_3_N_1D, then it is further raised to 225 mW cm^−2^ for (Co,Fe)_3_N_1C, and finally reaches 234 mW cm^–2^ by (Co,Fe)_3_N_2D. Moreover, the originally arced discharge polarization curve is gradually straightened and becomes almost linear by the end of the second discharge. As for the charging curves, a positive influence is also observed in the current-density ceiling and a similar straightening trend is found. The step offset at 155 mA cm^−2^ observed in (Co,Fe)_3_N_R is visibly weakened for (Co,Fe)_3_N_1D and further reduces for both (Co,Fe)_3_N_1C and (Co,Fe)_3_N_2D. Despite the elevations, the matured catalyst demonstrates increased slopes in both of its charge and discharge polarization curves, which may be triggered by the negative kinetic influence of maturation. To validate the observations, air electrodes at selected states were transferred and examined freshly in a three-electrode system. Similar optimization of ORR/OER bifunctionality (Fig. 2d), as well as increment in electrochemical double-layer capacitance (Supplementary Fig. 6), can be observed, in which the bifunctionality is defined by the voltage difference between ORR and OER branches typically collected at the current density of −2 mA cm^−1^ for ORR and 10 mA cm^−1^ for OER as compared in Supplementary Table 1, while Tafel slopes in Fig. 2e reflect easing kinetics despite slight rebounds in the latter two stages. On the basis of these electrochemical behaviors, it is preliminarily speculated that the transformation of (Co,Fe)_3_N during maturation in the initial cycles is a “double-edged sword,” which increases bifunctionality and availability of catalytic sites, but slightly passivates the overall electrocatalytic kinetics.

**Fig. 3:**
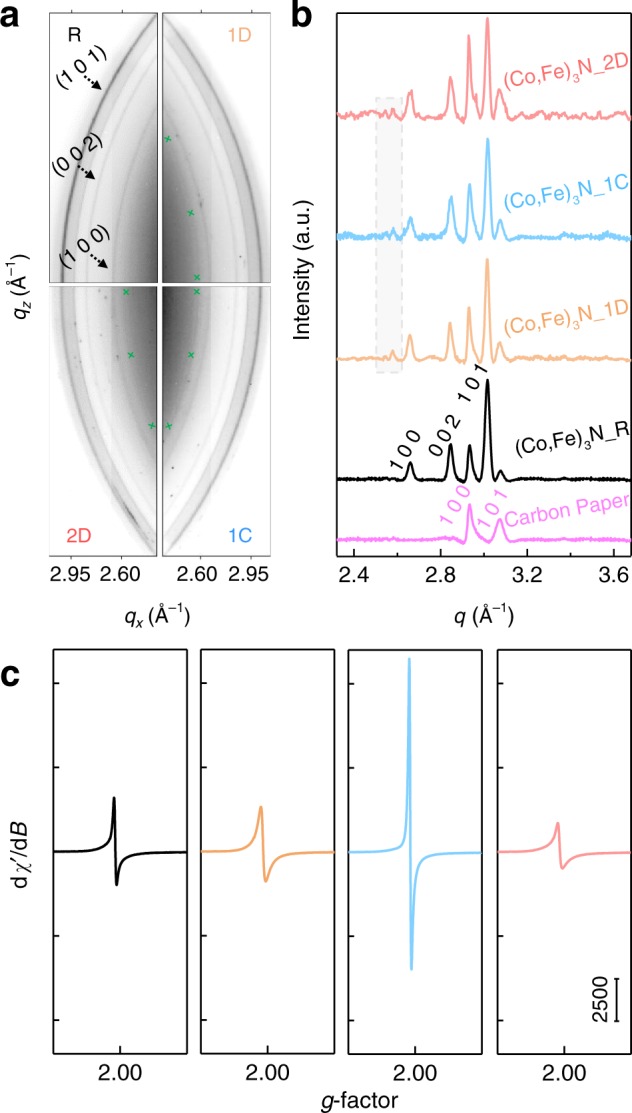
The crystal and electronic structures. **a** 2D WAXS images, **b** integrated SXRD patterns, including blank carbon paper, and **c** X-band EPR spectroscopies of electrocatalysts obtained at different electrochemical stages.

To investigate the structural evolution, two-dimensional wide-angle X-ray scattering (WAXS) patterns of the electrocatalysts at different stages were collected in Fig. 3a. The original rings of (Co,Fe)_3_N_R and blank carbon paper are generally maintained after electrochemical treatments, except for the two weak scattering rings at the respective *q* values of 2.58 and 2.54 Å^−1^. The synchrotron X-ray diffraction (SXRD) patterns in Fig. 3b further validate the emergence of a new phase, which can be indexed to the feature diffractions of hexagonal CoOOH (PDF#14-0673) as shown in Supplementary Fig. 11a ^38^. Fe incorporation is also considered here, so the new phase is noted as (Co,Fe)OOH. As a widely recognized insulator, the generation of the low-conductivity oxyhydroxide should be the reason for the easing kinetics observed in the electrochemical measurements^39^. Along with the emergence of the new diffractions, another evolution is observed in peak intensity. When setting the graphitic (1 0 0) facet of carbon paper as a reference, a gradual decrease is reflected in peak intensity of the nitride featured (1 0 1), which is diagnosed as partial vanishing of the original feature as a result of the phase transformation.

The variations in the electronic state of the associated metal species are reflected in its resonant feature as observed by electron paramagnetic response (EPR). Specifically, the broad resonance peak at *g* ≈ 5 (Supplementary Fig. 7), commonly associated with EPR-active Co^II^ (3*d*^7^) or Fe^III^ (3*d*^5^)^40,41^, first experiences an intensity increment when the battery is discharged, then falls at charge, and increases again at the second discharge. The Co^II^ emergence upon discharging is in agreement with the previous observation in ORR scans^26^. A reverse trend is observed with the free-electron resonance signal located at *g* = 2.0 in Fig. 3c, which exhibits an intensity maximum at (Co,Fe)_3_N_1C. Considering the EPR-silent feature of Co^III^ (3*d*^6^) and Fe^VI^ (3*d*^4^), the EPR signal fluctuation is ascribed to the temporal existence of Co^IV^ (3*d*^5^) and the corresponding formation of oxygen vacancies^40–42^. Besides, the intensity difference between the two discharge stages confirms an irreversible weakening of the nitride feature from continuous electrochemical control.

**Fig. 4:**
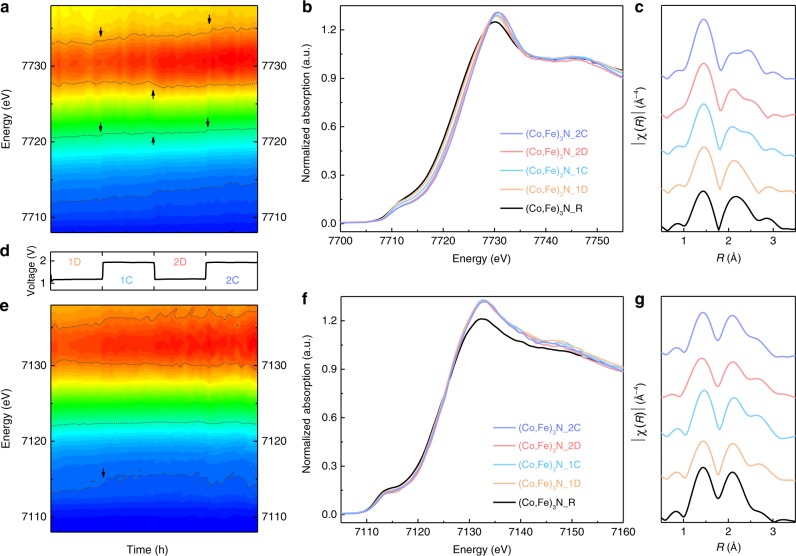
Operando X-ray absorption spectroscopic analysis. The operando XANES contour maps of **a** Co and **e** Fe K edge, and the corresponding **d** voltage profile in the first two cycles; the red and blue contours, respectively, represent high and low adsorption intensities. Operando XANES and the *k*^3^-weighted FT spectra of **d**, **e** Co and **f**, **g** Fe K edge at different electrochemical stages.

To monitor the evolution of the two metal ions, operando X-ray absorption spectra of the electrocatalyst during the first two discharge–charge cycles (Fig. 4d) were collected. The XANES contour map of Co is displayed in Fig. 4a and Supplementary Fig. 8a, which shows four continuous and distinguishable periods. As the battery operation begins, a slow shift to higher energy of Co adsorption edge position is indicated by the characteristic contour line near 7720 eV in the first discharge. In the ensuing charging process, a step rise occurred at the very beginning, followed by steady increment of the edge position along with right shift and intensity enhancement of the white-line peak. Then, when initiating the second discharge, a small shift to lower energy is observed for both edge and main peak; nevertheless, the overall ascent sustains. Last, when the process reaches the second charge (2C), another step rise appears, followed by stabilization of the edge position and the white-line peak intensity at the highest level. After completion of the two cycles, an overall 1.5-eV increase in the edge position is measured. This final edge position overlaps with CoOOH reference (Supplementary Fig. 11b), indicating the presence of Co^III^ feature in (Co,Fe)_3_N_2C. Based on XANES spectra (Fig. 4b), it can be stated that the valence state of Co continues to increase during the entire maturation process, which contradicts against the intensity fluctuation observed in EPR results. This conflict is due to the observed discrepancies in the two analytical techniques, as XAS generally includes all signal contributions and monitors the average oxidation change^36^. When taking these electrochemically inaccessible Co into consideration, it would counteract the generated Co^VI^ in the shell to give an average Co^III^ feature at the charged state. Another noticeable evolution is the gradual fading of the pre-edge peak at 7712 eV, implying a weakening nitride feature as well as increasing Co occupation at dipole-forbidden octahedral sites in oxyhydroxide, which is responsible for the continuous increment of average Co oxidation. According to previous studies based on a three-electrode system, oxyhydroxide generation in OER always occurs near the catalyst surface^19,24,25,43^. An analogous situation is also considered herein, that is, the interfacial Co is electrochemically accessible, while the bulk Co is isolated. This consideration is fully supported by X-ray photoelectron spectroscopy (XPS) spectra at different depths in Supplementary Fig. 9, showing the absence of nitride feature at the surface and current-driven shifts of surficial elements (Supplementary Note 1)^44^. Combined with the EPR results, Co^II^ is identified in oxyhydroxide shell upon discharge, while Co^IV^ exists after the battery is charged. Figure 4e, f and Supplementary Fig. 8b display operando Fe K-edge XANES contour map. Progressive reductions of the pre-edge peak intensity at 7114 eV and smooth increase of the white-line peak intensity are observed and complete at the beginning of the first charge. These changes are also a result of surficial transformation from nitride to oxyhydroxide. Other than that, its constant edge position at ~7123 eV is diagnostic of the stable Fe^III^ feature in the cycling process.

The electrochemical influence on the coordination environment of metal ions is also captured using operando extended X-ray absorption fine structure (EXAFS). Co K-edge *k*^3^-weighted Fourier-transformed (FT) results are shown in Fig. 4c. As the base spectrum, two major peaks centered at 1.4 and 2.1 Å are observed for (Co,Fe)_3_N_R, respectively, representing the interatomic distance of Co–N and Co-metal shells in nitrides^27^. Comparatively, a slight right shift and intensity elevation of the Co–N peak is found in (Co,Fe)_3_N_1D, while the peak of Co-metal shell reduces in intensity. These changes are symptoms for initiating oxyhydroxide formation and overlapping of Co–N with Co–O shell at 1.4 Å. A new peak appears in (Co,Fe)_3_N_1C at 2.5 Å, which can be assigned to the typical Co-metal shell in Co-containing oxyhydroxide as shown in Supplementary Fig. 11c ^43,45^. This new peak experiences slight position swings in the ensuing cycling process (Supplementary Fig. 10), corresponding to changes in the interatomic distance induced by valence variations of Co in oxyhydroxide^43^. Specifically, when the catalyst is charged, high-valence Co constrains its distance with neighboring metals or oxygens and causes left shift of the corresponding peaks. This maturation also causes the ascending of Co–O coordination number suggested by its increasing Co–O/N intensity. Regarding Fe K-edge *k*^3^-weighted FT results, Fig. 4g compares spectra obtained at each stage. As the feature Fe-metal distance in oxyhydroxide, a peak at 2.6 Å stands out in (Co,Fe)_3_N_1D as an evidence for oxyhydroxide generation. Aside for this variation, the spectra experience no significant changes in the following process.

**Fig. 5:**
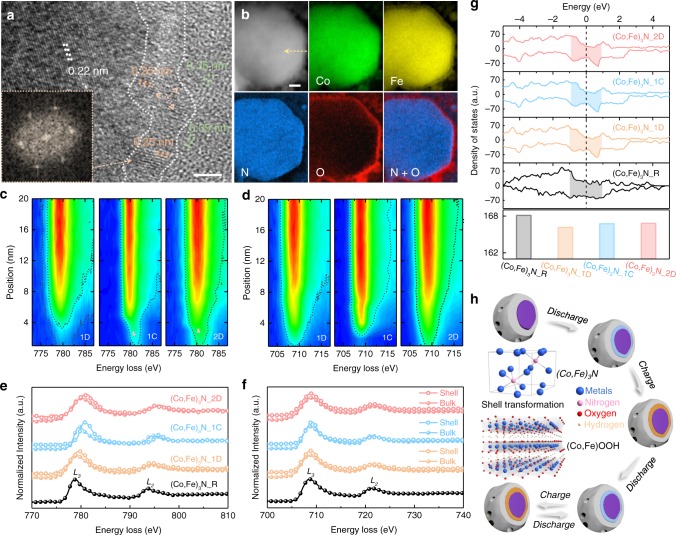
The local chemical state changes and schematic of maturation process. **a** HRTEM image at the edge region of a (Co,Fe)_3_N_2D particle showing three regions with distinguishable lattice fringes; scale bar: 2 nm. The inset shows the fast FT (FFT) pattern of the intermediate oxyhydroxide region. **b** EELS elemental mapping of a (Co,Fe)_3_N_2D particle; scale bar: 10 nm. **c** Co, **d** Fe L-edge energy-loss near-edge structure (ELNES) contour maps (the red and blue colors represent high and low intensities), and **e**, **f** their corresponding ELNES curves along the marked arrows crossing the shell and bulk regions as marked in Supplementary Fig. 11a, b and **b**. **g** Density-functional theory (DFT)-calculated DOS of electrocatalysts at different electrochemical stages based on the computational models in Supplementary Fig. 13. **h** Schematic illustration for the maturation pathway of (Co,Fe)_3_N_R with shell transformation from nitride to oxyhydroxide during cycling. The blue and orange regions both represent the oxygen intermediate layers, while the two colors demonstrate their different chemical states at discharge or charge.

All the above characterizations point toward the fact that the surficial metal nitride experiences continuous transformation during cycling, but isolated analysis of the shell region is still required to open the “black box” and directly visualize the maturation process. As such, several representative particles at different electrochemical stages were selected for TEM and electron energy-loss spectroscopy (EELS) analyses. When compared with (Co,Fe)_3_N_R (Fig. 1c), HRTEM image of (Co,Fe)_3_N_2D in Fig. 5a reveals the presence of a new intermediate layer with distinguishable crystal information between the nitride bulk and carbon coating layer. In this new layer, typical hexagonal lattice fringes are observed with a *d* spacing of 0.25 nm, and its hexagonal symmetry is further verified by the fast FT pattern. Except for these variations, the bulk metal nitride and the carbon layer are preserved after battery operation. The presence of this new layer is further reflected by a sharp O-rich and N-deficient boundary with a thickness of ~4 nm (Fig. 5b and Supplementary Fig. 12), which is in agreement with the XPS analyses at different depths (Supplementary Fig. 9 and Note 1). These results confirm the generation of oxyhydroxide intermediate layer starting at the initial discharge (Supplementary Fig. 12a, c), which maintains a similar thickness for both (Co,Fe)_3_N_1C (Supplementary Fig. 12b, d) and (Co,Fe)_3_N_2D (Fig. 5b and Supplementary Fig. 12e). Another key point revealed is the relatively higher Fe content in the intermediate layer, which is explained by its lower electrochemical stability and supported by the completion of Fe pre-edge weakening at the very beginning of the first charge in operando XANES.

Ex situ Co and Fe L-edge EELS analyses are conducted to locally decipher their chemical evolutions. Two key criteria are considered, including the peak energy position and the intensity ratio of L_3_/(L_2_ + L_3_), or commonly stated as L_3_ branch ratio^46,47^. The contour maps in Fig. 5c, d display the electron energy-loss near-edge structure spectra (Supplementary Fig. 13a, b) acquired along the labeled arrows in Fig. 5b and Supplementary Fig. 12a, b. As shown, the shells of (Co,Fe)_3_N_1C and (Co,Fe)_3_N_2D exhibit a 1- and 0.7-eV right shift in their Co L_3_ position, respectively (Fig. 5e, Supplementary Fig. 13a, and Table 2), demonstrating their clear chemical differences in the shell and bulk regions. As for Fe L_3_ peak position (Fig. 5d, f and Supplementary Fig. 13b), it remains relatively constant along the arrows. Besides, considering the comparable bulk concentration of Co and Fe, the color difference in the shell reflects increasing Co concentration in the generated oxyhydroxide during maturation. Focusing on the shell region within the first 4 nm, Fe shows significantly higher intensity than Co in (Co,Fe)_3_N_1D, suggesting the dominant role of Fe in preliminary oxyhydroxide. By the time the catalyst reaches (Co,Fe)_3_N_2D, the Co signal is visibly strengthened and coexists at a similar intensity with Fe. The cation rearrangement in oxyhydroxide layer during maturation is led by a dissolution/redeposition mechanism^24^. To quantify the increasing Co participation, the average Co/Fe ratios in oxyhydroxide layer were calculated based on EELS line scans to be 0.68 for (Co,Fe)_3_N_1D, 0.87 for (Co,Fe)_3_N_1C, and 0.93 for (Co,Fe)_3_N_2D. This composition rearrangement should be responsible for the small kinetic recovery in electrochemical measurements. To validate this causality, the density of states (DOS) for the four cycling states are calculated based on models in Supplementary Fig. 14 and compared in Fig. 5g. Besides the metallic feature of nitrides revealed by their continuous DOS at the Fermi level, the influences of oxyhydroxide generation and increasing Co participation on electrical conductivity are also identified^27^. The highest DOS of (Co,Fe)_3_N_R near the Fermi level demonstrates the passivation effect of the generated oxyhydroxide shell. When compared with the preliminary states of (Co,Fe)_3_N_1D, the later two states of (Co,Fe)_3_N_1C and (Co,Fe)_3_N_2D are slightly more intense, suggesting increased carrier concentration after the cation rearrangement. Therefore, the theoretical evidence is provided for the electrochemical kinetic changes.

L_3_ branch ratios were also calculated after background subtraction as plotted in Supplementary Fig. 13c and Table 2^48^. For the bulk region, a continuous downward trend of L_3_ branch ratio is observed in Co L-edge spectra, which implies slight increase in average valence states during maturation. It should be noted that these bulk spectra represent the average chemical states due to the transmission-probing manner of EELS, and therefore this tendency matches well with operando XANES results. However, a maximum valence is acquired at the shell region of (Co,Fe)_3_N_1C that is even higher than the reported Co_3_O_4_ reference^48^, which then falls back in (Co,Fe)_3_N_2D to a similar level as (Co,Fe)_3_N_1D. Due to the ex situ and high-vacuum TEM environment, the absolute valences of shell elements are relatively lower than their actual states in battery operation, but the shell-bulk difference and variation trend are still detected. By combining with the above analyses, the prediction on shell-bulk-type configuration and periodic valence swings of surficial Co between Co^II^ and Co^IV^ during cycling is confirmed. Regarding Fe in oxyhydroxide intermediate layer, its L_3_ ratios remain constant at a narrow range close to Fe^III^^49^. Its feeble response to the electrochemical control stands with the XAS results, revealing the different roles of the two metals.

## Discussion

As illustrated in Fig. 5h, the dynamic picture of metal nitride as bifunctional oxygen electrocatalyst in Zn–air battery operation is presented. Briefly, the as-prepared (Co,Fe)_3_N_R is equipped with hierarchical morphology, optimal pore tunnels, and high electrical conductivity, so that the outstanding electrochemical parameters are endowed. The maturation pathway launches immediately upon the initial discharge. The primary effect of this stage is generation of the preliminary oxyhydroxide shell, which is accompanied by slight reduction of nitride features and emergence of Co^II^ and Fe^III^ features. It suggests the separation in trajectories of the shell and bulk regions, but this configuration is not yet matured and Co-related evolution is still in progress. Next, high-valence Co^VI^ emerges ostensibly in the 1C process, along with the appearance of oxyhydroxide-featured Co-metal interatomic shell in operando EXAFS spectra. Comparatively, the negligible change is demonstrated in bulk Co. The Co participation in oxyhydroxide shell is further aggravated in 2D, which ends when contradiction of increasing the overall Co valency and falling surficial Co valence state is observed. At this stage, the maturation of the as-prepared metal nitride is completed, and the battery parameters stabilize. The large performance enhancement in maturation is ascribed to active site transformation and the corresponding expansion in the electrochemical active area provided by oxyhydroxide shells. Nevertheless, as supported by theoretical calculation, maturation is not a process without any downside. The low conductivity of this new phase does slightly passivate the overall electrocatalytic kinetics. For the following cycles, although this shell-bulk configuration is maintained, periodic swings of the Co valence state will continue to occur within the oxyhydroxide shell.

At this point, with bimetal nitride as a general representation, a new recognition of dynamic electrocatalyst and its corresponding maturation process in rechargeable Zn–air battery are thoroughly presented. There are several points that require clarification. First, physical factors such as air circulation and electrolyte permeation on carbon paper or Zn plate are excluded as origins for performance improvement due to the fading of noble-metal reference battery within the same conditions. Hence, the change in electrochemical performance is solely linked to the phase transformation of metal nitrides during the battery operation. Second, the oxyhydroxide shell in the matured configuration exhibits a hexagonal crystalline that significantly differs from the amorphous feature in the previous report^24^. Its stable surface chemistry prevents further transformation after maturation and constrains the shell thickness to ~4 nm. This phenomenon combined with the settled battery parameters suggests termination of the lattice oxygen activation process^25^. Third, several previous reports have proposed to combine the conversion reactions of principal elements with the oxygen reactions to form hybrid batteries^50–52^. From the above results, it is clear that the performance contribution of Co redox is too small to be reflected by any additional voltage plateaus, especially in long-period cycling tests. The periodic swings in the oxyhydroxide shell should be understood as an inherent process of galvanostatic cycling, and the oxygen-related redox is the sole dominator for the battery behavior. Fourth, in this study, Co in the intermediate (Co,Fe)OOH layer is considered as the primary performance contributor due to its periodic variations during the electrochemical process, while Fe remains relatively idle. Although different pictures may be presented in nitrides with other metal combinations, the unveiled maturation and the current-driven configuration is pervasive for them. Moreover, owing to the similar chemical reactivity, such process is also applicable for most of metal-based electrocatalysts in Zn–air battery, such as metals, alloys, oxides, phosphides, and chalcogenides^20–22,24,25^. Finally, by gathering those fundamental understandings of electrocatalyst within battery operation, material design principles can be optimized accordingly. As for an ideal electrocatalyst for rechargeable Zn–air battery, two key components should be possessed besides rational geometrical optimization. One is a thin and highly active oxyhydroxide shell that serves as the primary activity contributor responsible for catalyzing the oxygen reaction in battery cycling, and another is a highly conductive bulk to maintain the electrochemical kinetics.

In summary, a bimetal nitride with secondary morphology and hierarchical porosity has been synthesized. When served as the electrocatalyst in Zn–air battery, it not only delivers performance similar to precious benchmarks at its native stage, but also demonstrates a maturation process that leads to a maximum power density of 234 mW cm^−2^ and a discharge–charge voltage gap of 0.85 V at 30 mA cm^−2^ that lasted for over 300 h. This maturation process is reproduced in a long-period cycling test, which achieves a voltage gap of 0.64 V at 5 mA cm^−2^ for over 750 h of cycling. These electrochemical parameters make it one of the top-tier electrocatalysts reported in Zn–air battery studies as compared in Supplementary Table 3. More importantly, the underlying evolutions of metal nitrides upon battery operation are clearly visualized and explained using a wide range of characterization techniques. On the basis of the obtained evidences, a new concept of dynamic electrocatalyst is established with a current-driven shell-bulk configuration that is believed to be pervasive for most metal-based electrocatalysts. This mechanistic insight into the dynamic electrocatalyst paves a new avenue for future innovations on electrocatalyst designs regarding rechargeable Zn–air battery applications.

## Methods

### Synthesis

The preparation of bimetal nitride adopted herein is a modified method based on the literature^53^. It includes three major steps for an entire procedure. The first step is to synthesize the solid nanocuboid precursor. Typically, 3 g of polyvinylpyrrolidone (K30) with an average molecular weight of 40,000 was fully dissolved in 15 mL of anhydrous ethanol to form a colorless and viscous solution. In a separate container, 1.4 g of cobalt acetate tetrahydrate was dissolved in 85 mL of anhydrous ethanol. These two solutions were mixed within a 250-mL round-bottom flask and refluxed for 12 h at 90 °C under magnetic stirring of 1200 r.p.m. After centrifugation using ethanol at 7000 r.p.m. three times and sequential drying in air, the pink Co-containing precursors were obtained. Then, for the cation-carving process, 0.2 g of fresh Co-containing precursors were even dispersed in 20 mL of anhydrous ethanol under ultrasonication. Another clear solution is formed by dissolving 0.16 g of iron sulfate heptahydrate into 200 mL of 1:1 ethanol–water solution under nitrogen gas protection. The above two solutions were mixed and allowed to react under stirring at 500 r.p.m. for 15 min. Nitrogen gas was continuously bubbled during this process. The yellow powders, that is, the bimetal-layered double hydroxide, were collected through centrifugation at 7000 r.p.m. and washed with ethanol. Last, the ammonization process was calcined at a high temperature. Prior to that, the bimetal-layered double hydroxide was pretreated with dopamine hydrochloride polymerization within 0.01 M tris(hydroxymethyl)aminomethane buffer aqueous solution. The ratio between the bimetal-layered double hydroxide and dopamine hydrochloride was fixed to be 2, and the mixed solution was stirred at 500 r.p.m. for 5 h. Afterward, the dried black precipitate was collected by centrifugation at 9000 r.p.m. and washed with deionized water and ethanol. The precipitate was heat-treated at 500 °C under ammonia gas for 1 h, with a ramp rate of 2 °C/min. Argon gas was used to protect the sample during the ramping period, and the tube furnace was purged by ammonia gas only after the temperature has stabilized at 500 °C. After cooling down to room temperature, the targeted bimetal nitride of (Co,Fe)_3_N_R was obtained. The CoOOH references were prepared following the procedures in the previous report^54^.

### Characterization

The morphology and energy-dispersive spectra were collected using field-emission scanning electron microscope (UltraPlus FESEMs, 20 kV) and TEM (FEI Titan 80-300 LB, 300 kV) equipped with an X-ray spectrometer detector. The EELS was acquired by a double aberration-corrected TEM (FEI Titan 80-300 HB, 200 kV). The surface area and porous structure were calculated based on N_2_ isotherm obtained on a Brunauer–Emmett–Teller analyzer (Quantachrome Instruments QuadraSorb SI4), and the pore-size distribution was calculated based on the density-functional theory model. The structural information was obtained by XRD (Rigaku Ultima IV with Cu K*α* as the radiation source) and Raman spectroscopy (WiTEC alpha300R Raman microscope at an excitation line of 532 nm). The WAXS images were acquired in transmission mode at Brockhouse X-ray Diffraction and Scattering Beamlines (High Energy Wiggler, BXDS-WHE) of Canadian light source. The energy was set at 35 keV with a wavelength of 0.359106 Å. The 2D images were calibrated by a LaB_6_ standard sample. The General Structure Analysis System II software was employed to average and integrate the SXRD patterns^55^. The XPS was carried out using PHI 5000 VB III, and argon ion bombardment was used to remove the sample surface so that their bulk chemical information can be collected. The result was calibrated with a reference C *1s* peak set to 284.84 eV. The EPR was acquired at 77 K by a Bruker EMX-10/12 spectrometer (2-mW microwave power, 9.4-GHz microwave frequency, 100-kHz modulation frequency, and 0.2-mT modulation width).

Operando X-ray absorption spectroscopy was performed at Soft X-ray Microcharacterization Beamline (SXRMB, 06B1-1) of Canadian Light Source and a homemade Zn–air battery prototype was directly adopted as the operando setup. The back side of carbon paper without electrocatalyst was placed to face the incident photon and the detector. Both of Co (7709 eV) and Fe K-edge (7112 eV) spectra were recorded on fluorescence yield mode. Standard metal foils and metal oxides purchased from Sigma-Aldrich were used as a reference. The spin-polarized computations were conducted using Vienna ab initio simulation package^56,57^. The ion–electron interactions were described by the projector-augmented wave method^58^. The general gradient approximation in the Perdew–Burke–Ernzerhof form was applied^59,60^. The convergence criteria were, respectively, set to 0.03 eV Å^−1^ and 10^−5^ eV for the residual force and energy during the structure relaxation. The Brillouin zone is represented by the set of 3 × 3 × 1 and 5 × 5 × 1 *k* points for the geometry optimizations and DOS computations. To avoid the interaction between two periodic units, a vacuum space of 20 Å was employed. The integral DOS intensity between −1 and 1 eV was calculated as the basis to compare the theoretical electrical propoerty^27^.

### Electrochemical measurement

The Zn–air batteries were fabricated based on a homemade prototype^37^. Prior to battery fabrication, the electrocatalyst ink was prepared by dispersing the electrocatalysts in 0.2 wt% Nafion solution and then sprayed onto a carbon paper (SGL Carbon; Ion Power Inc.) with a loading of 1 mg cm^−2^ to form the GDL. This exact process was also used to prepare the performance benchmark with an equal amount of 20 wt% Pt/C and RuO_2_. Then, a polished zinc plate, separator, and GDL were assembled layer by layer to form Zn–air battery, which was filled with 6 M potassium hydroxide and 0.2 M zinc acetate aqueous solution as an electrolyte. The cycling tests were carried out using galvanostatic method on a Land battery tester (LAND-CT2001A, Land Electronic Co., Ltd., Wuhan). The GDLs were treated to the predetermined electrochemical states, and then taken out immediately for these ex situ characterizations. Their polarization curves were measured on a Gamry 5000E workstation. The bifunctional electrocatalytic measurements were performed on a Biologic VSP 300 workstation using a three-electrode system, in which GDLs were used directly as the working electrode, while a saturated calomel electrode and a graphitic rod served as the reference and counter electrode. Oxygen-saturated 0.1 M potassium hydroxide aqueous solution was used as the electrolyte. The obtained data were calibrated to the reversible hydrogen electrode with the Nernst equation and further converted to the overpotential representing the difference against E°(O_2_/H_2_O) at 1.23 V. The Tafel slopes were calculated according to the Tafel equation.

## Supplementary information

Supplementary Information

## Data Availability

The data that support the findings of this study are available from the corresponding author upon reasonable request.
