# Supplementary material for: Dynamic electrocatalyst with current-driven oxyhydroxide shell for rechargeable zinc-air battery
Source: Nat Commun. 2020 Apr 23;11:1952. doi: 10.1038/s41467-020-15853-1 (PMC7181633; doi:10.1038/s41467-020-15853-1)
Supplement: Supplementary file 1 — Supplementary Information [file 41467_2020_15853_MOESM1_ESM.pdf]

Supplementary Information

Dynamic electrocatalyst with current-driven oxyhydroxide shell for Rechargeable zinc-air battery

Deng *et al.*

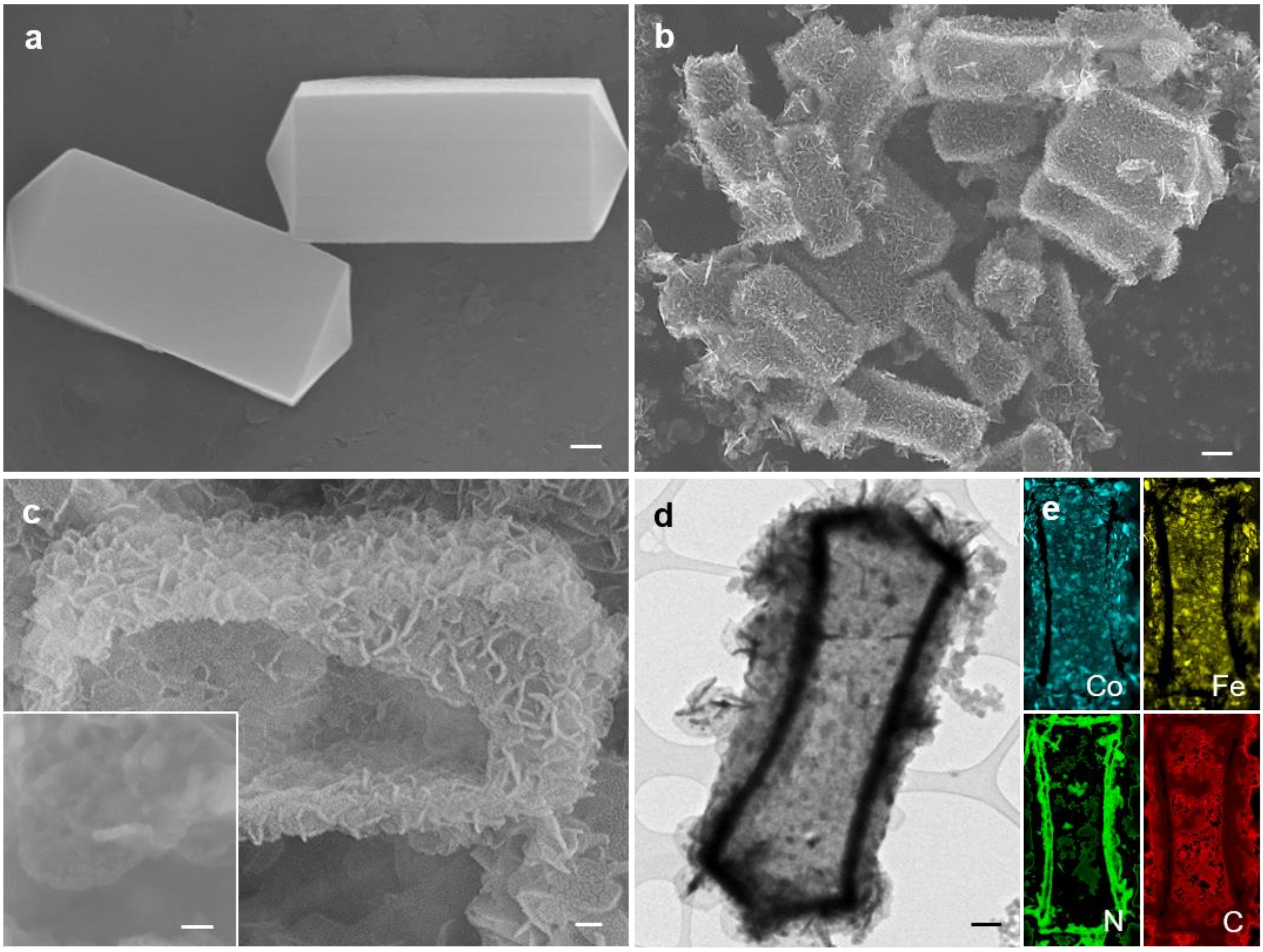

Supplementary Fig. 1 | Morphology and elemental distribution. Scanning electron microscopy images of (**a**) Co-containing precursor and (**b**, **c**) secondary nanocuboids at different magnification, inset of (**c**) shows the enlarged image of a primary nanosheet. The nanosheets have lengths of around 100 nm and show degrees to porosity. (**d**) Transmission electron microscopy image and (**e**) the corresponding elemental mappings. Scale bar: (**a**) 200 nm, (**b**) 500 nm, (**c**, **d**) 100 nm and inset of (**c**) 50 nm.

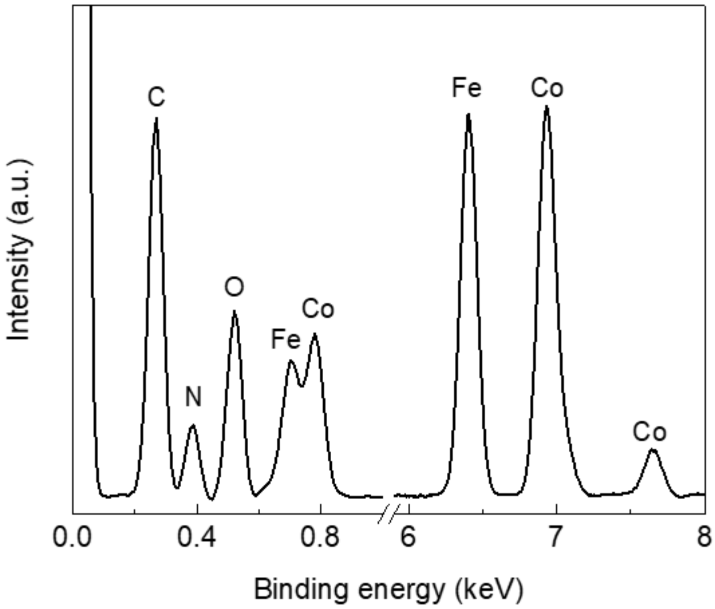

Supplementary Fig. 2 | Energy-dispersive spectrometer curve of $(Co,Fe)_3N$_R.

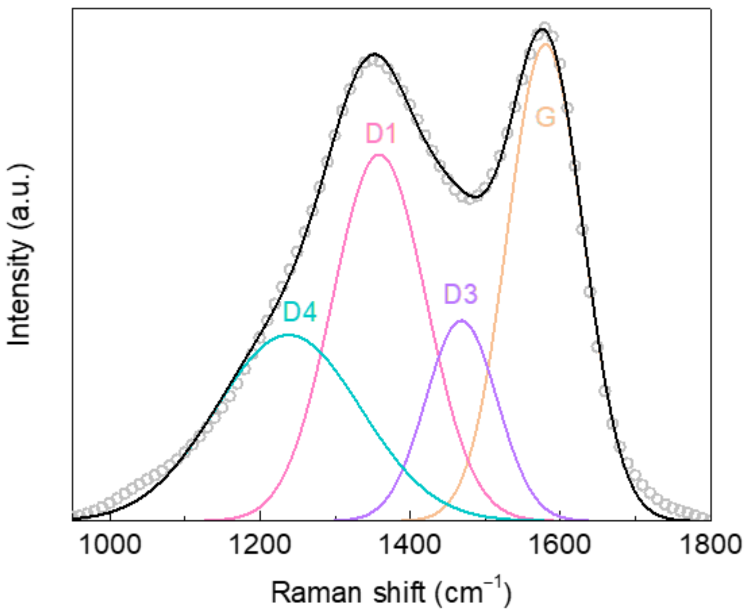

Supplementary Fig. 3 | Raman spectra of (Co,Fe)$_3$N_R. It is deconvoluted into four major peaks, including G band reflecting defect-free *sp*^{2} carbon with E_{2g} symmetry, D1 band representing disorder A_{1g} symmetry of graphite, D3 band attributed to *sp*^{3} amorphous carbon and the D4 band of polyene-like structure. As the basis for defect degree of carbon networks, I_{D1}/I_G is calculated to be 0.95 for (Co,Fe)$_3$N_R.[1]

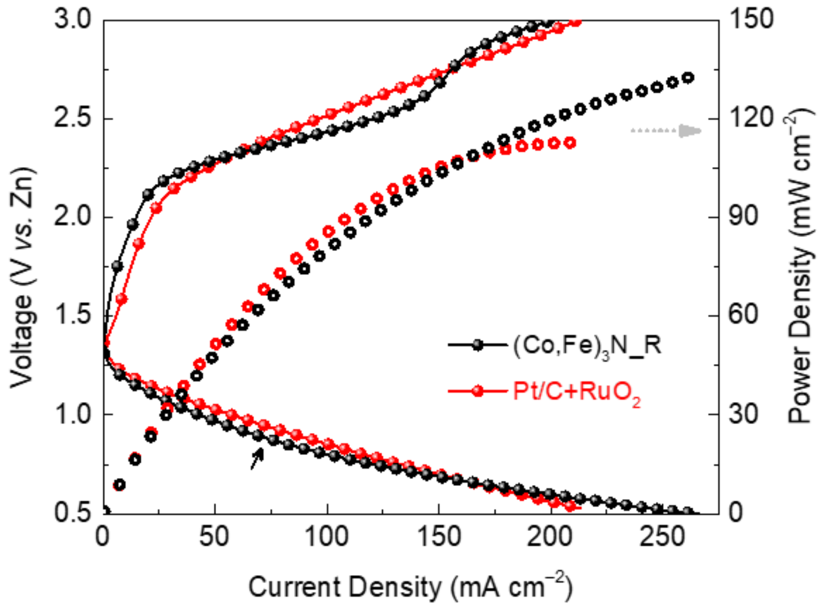

Supplementary Fig. 4 | Polarization curves and power density plots of Zn-air batteries.

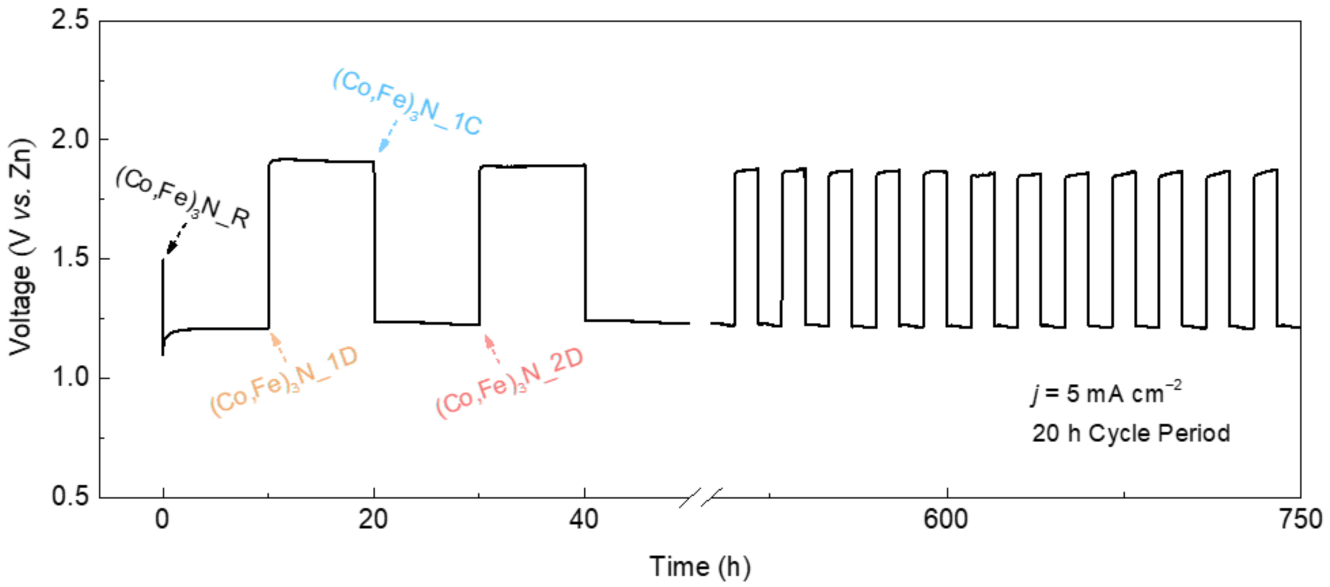

Supplementary Fig. 5 | 20 hours cycling profile under a current density of 5 mA cm^{-2}. The states of electrocatalysts are labelled as raw $(Co,Fe)_3N$_R, after initial discharged $(Co,Fe)_3N$_1D, after initial charged $(Co,Fe)_3N$_1C, and after second discharged $(Co,Fe)_3N$_2D.

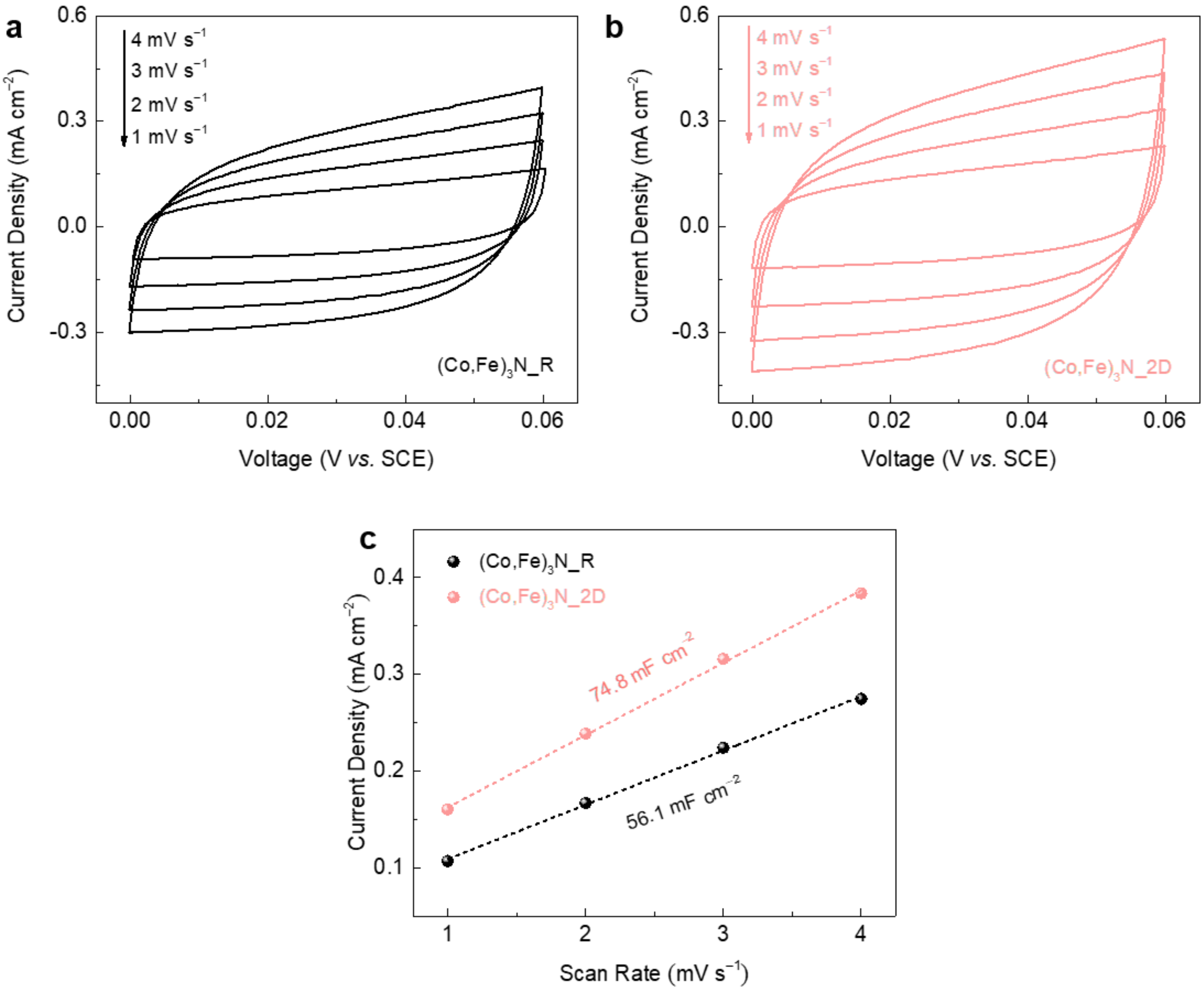

Supplementary Fig. 6 | Cyclic voltammetry (CV) curves and double-layer capacitance (C_{dl}). (**a, b**) CV curves of $(Co,Fe)_3N$_R and $(Co,Fe)_3N$_2D measured in 0.1 M KOH electrolyte at the scan rates of 1 to 4 mV s^{-1} within a non-faradic voltage window. (**c**) Current density at the potential of 0.03 V (*vs.* SCE) as function of the scan rate. The linear fitting slopes are used to determine their respective C_{dl} that corresponds with the electrochemical active surface area.

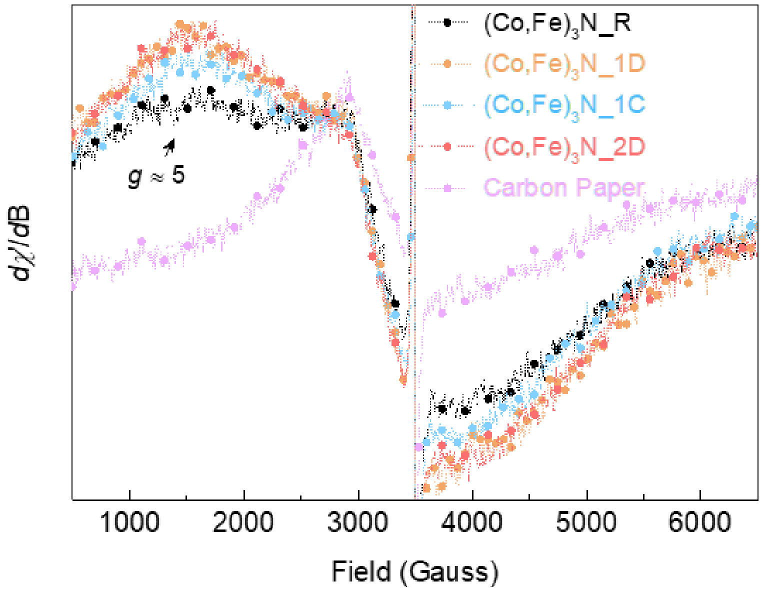

Supplementary Fig. 7 | X-band electron paramagnetic response spectroscopies of electrocatalysts at different electrochemical stages with carbon paper as a blank reference.

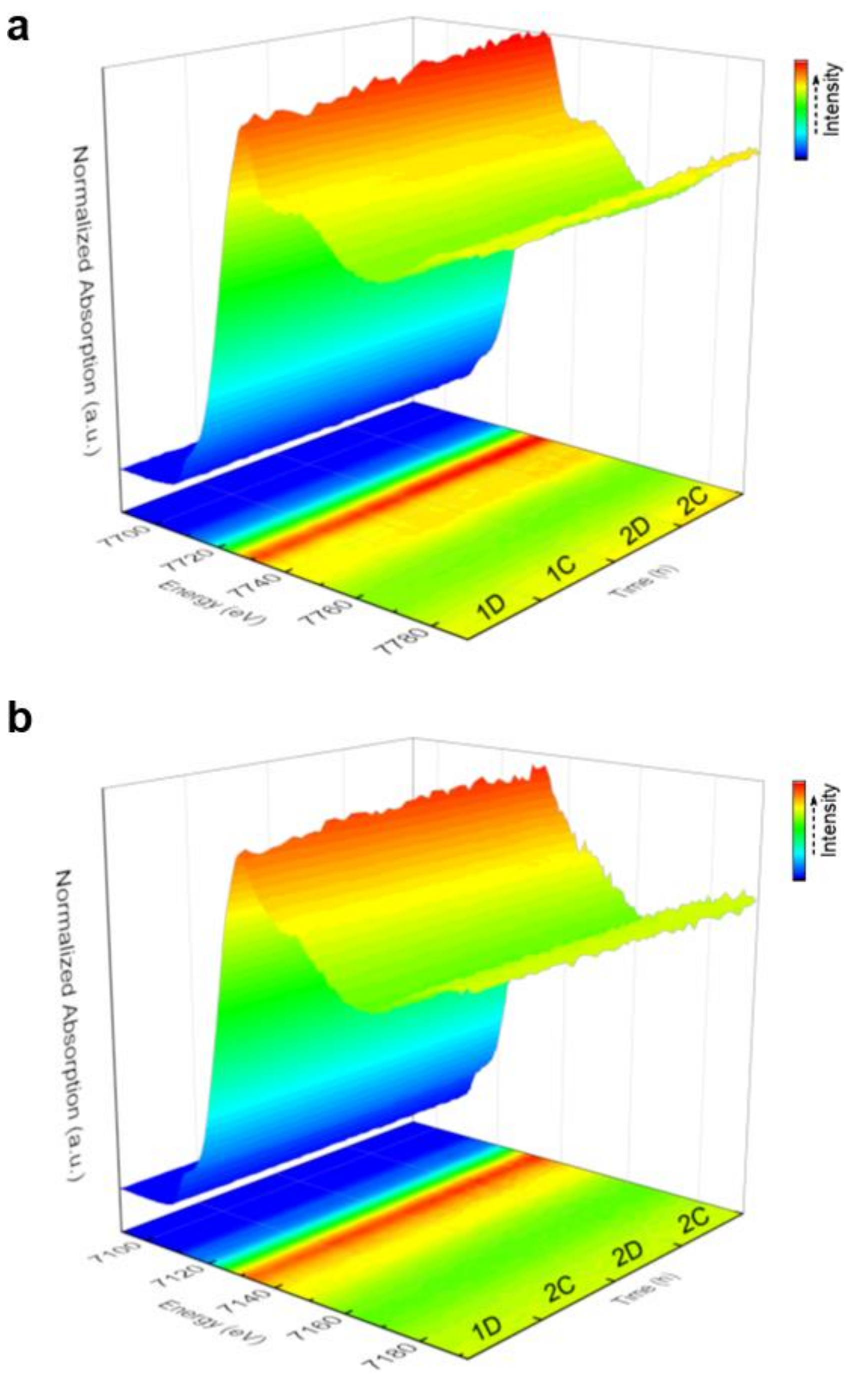

Supplementary Fig. 8 | *Operando* X-ray adsorption near-edge structure spectra and corresponding contour projections.

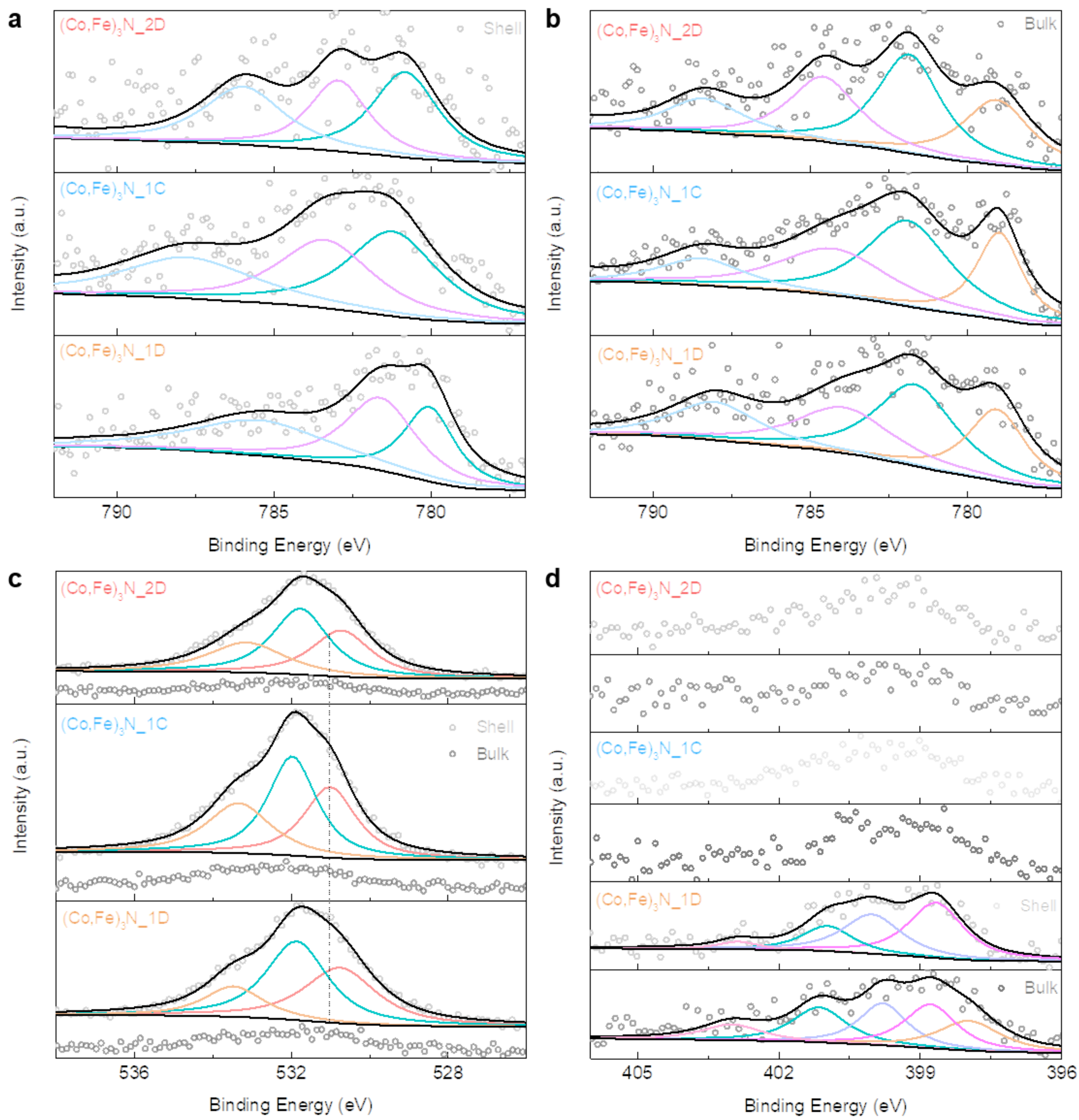

Supplementary Fig. 9 | High-resolution X-ray photoelectron spectroscopy (XPS) spectra. (**a**, **b**) Co *2p$_{3/2}$*, (**c**) O *1s* and (**d**) N *1s* collected from surface or bulk. The bulk XPS information were collected after surficial Ar^+ etching for 20 nm.

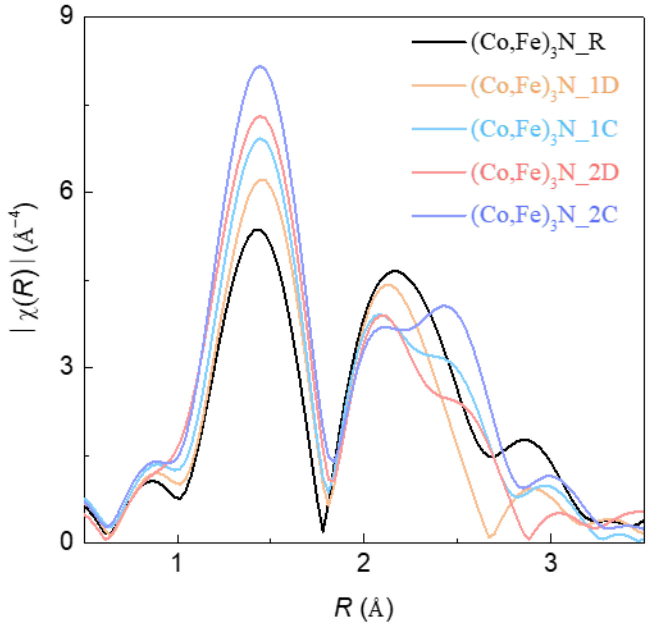

Supplementary Fig. 10 | Co K-edge k^3-weighted Fourier transform (FT) spectra of electrocatalysts at different electrochemical stages.

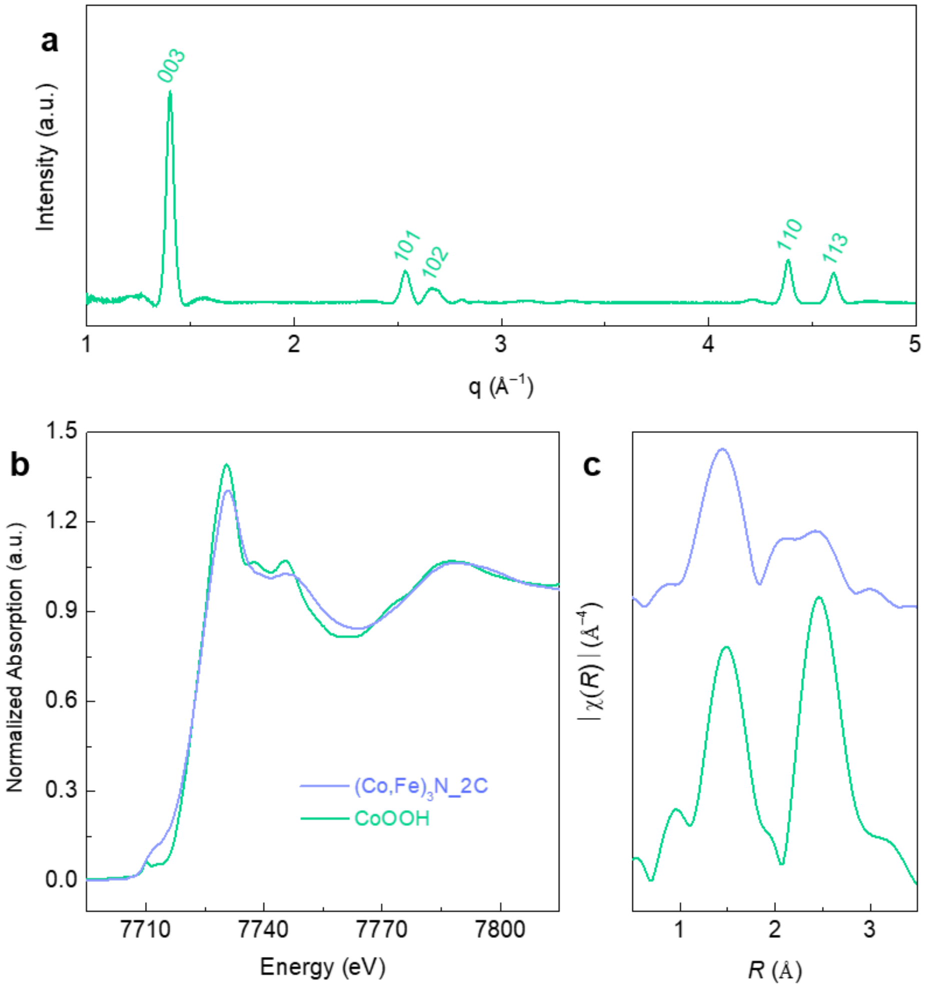

Supplementary Fig. 11 | Structural and chemical characterization of CoOOH. (**a**) Synchrotron X-ray diffraction pattern of CoOOH with a hexagonal layered structure (PDF#14-0673). (**b**) Co K-edge X-ray adsorption near-edge structure and (**c**) k^3-weighted FT spectra comparison between CoOOH with **(**Co,Fe)$_3$N_2C.

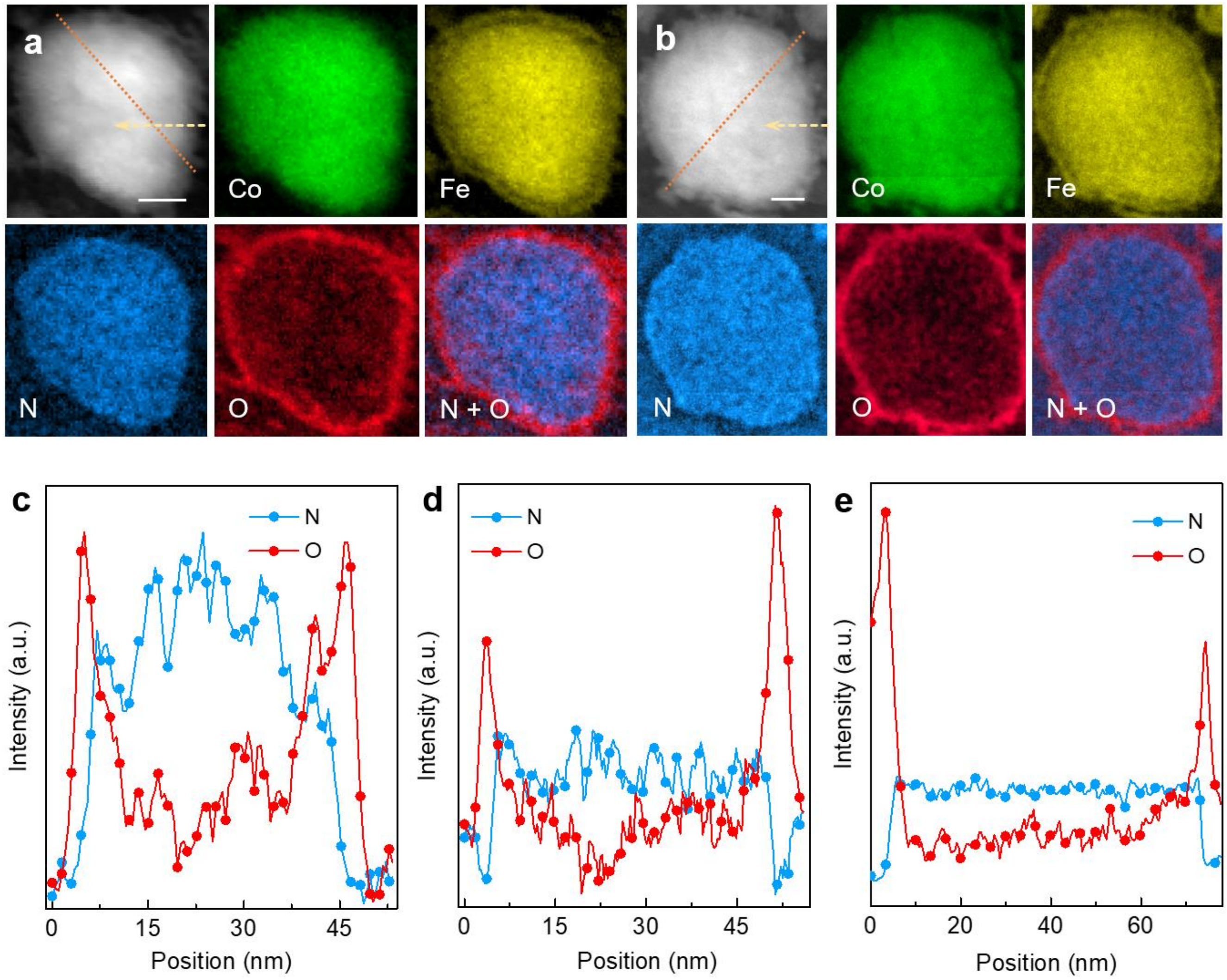

Supplementary Fig. 12 | The electron energy loss spectroscopy elemental mappings and line scans. (**a**, **c**) $(Co,Fe)_3N$_1D, (**b**, **d**) $(Co,Fe)_3N$_1C and (**e**) $(Co,Fe)_3N$_2D. Scale bar: (**a**, **b**) 10 nm.

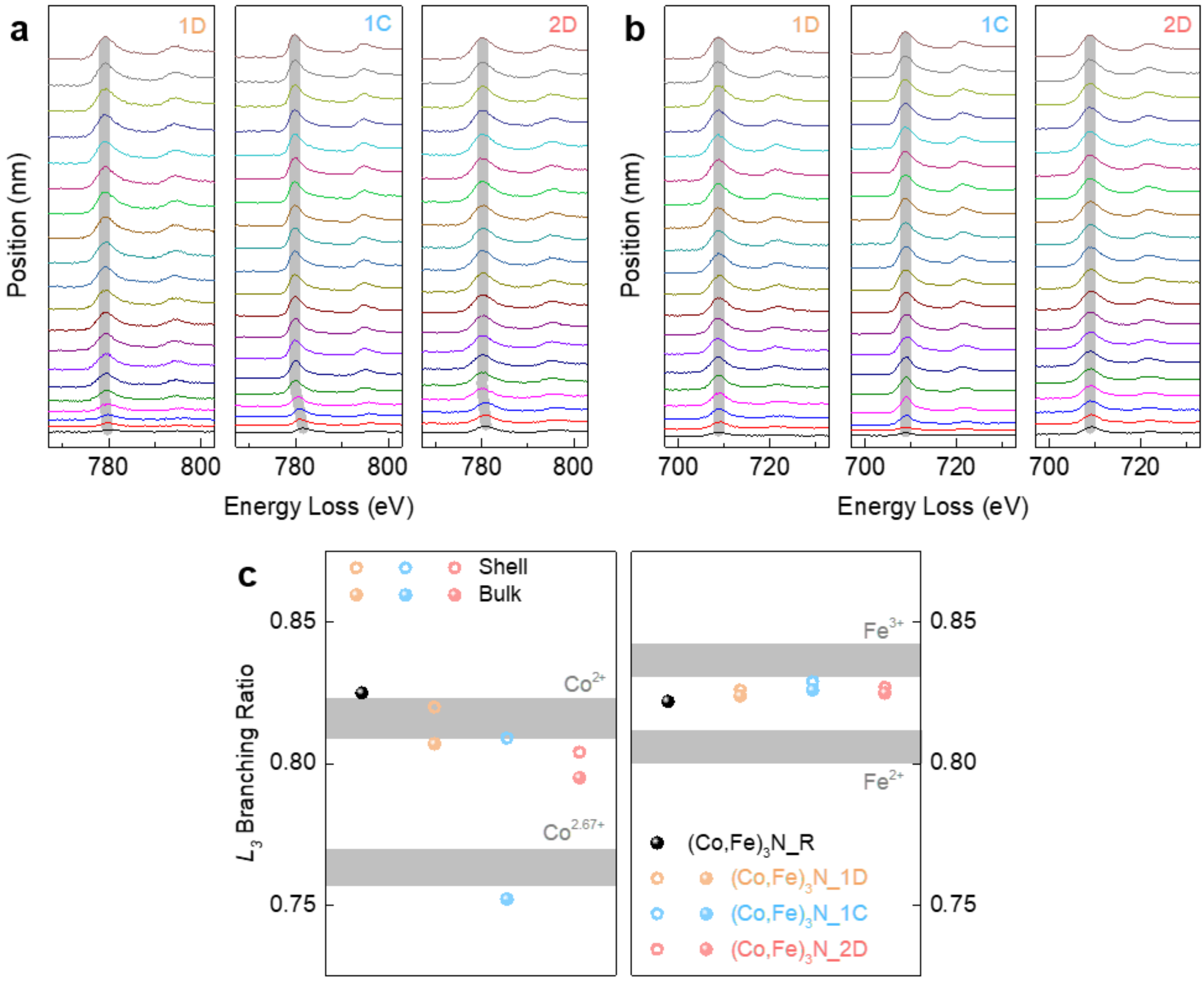

Supplementary Fig. 13 | The electron energy-loss near-edge structure (ELNES) analyses. (**a**) Co and (**b**) Fe L-edge ELNES spectra along the arrows marked in Supplementary Fig. 12a, b and Fig. 5b; (**c**) the L_3 branching ratio at different electrochemical stages, in which the reference location of valence states are based on literatures.[2,3] Before ELNES spectra processing, all the background intensities of Co and Fe L-edge were subtracted by a step function of Arctan.[2] The L_3 branching ratio describes the variation between the L_3 and L_2 white-line peaks, and it is defined by the intensity ratio of $I_{L3}/(I_{L3}+I_{L2})$.

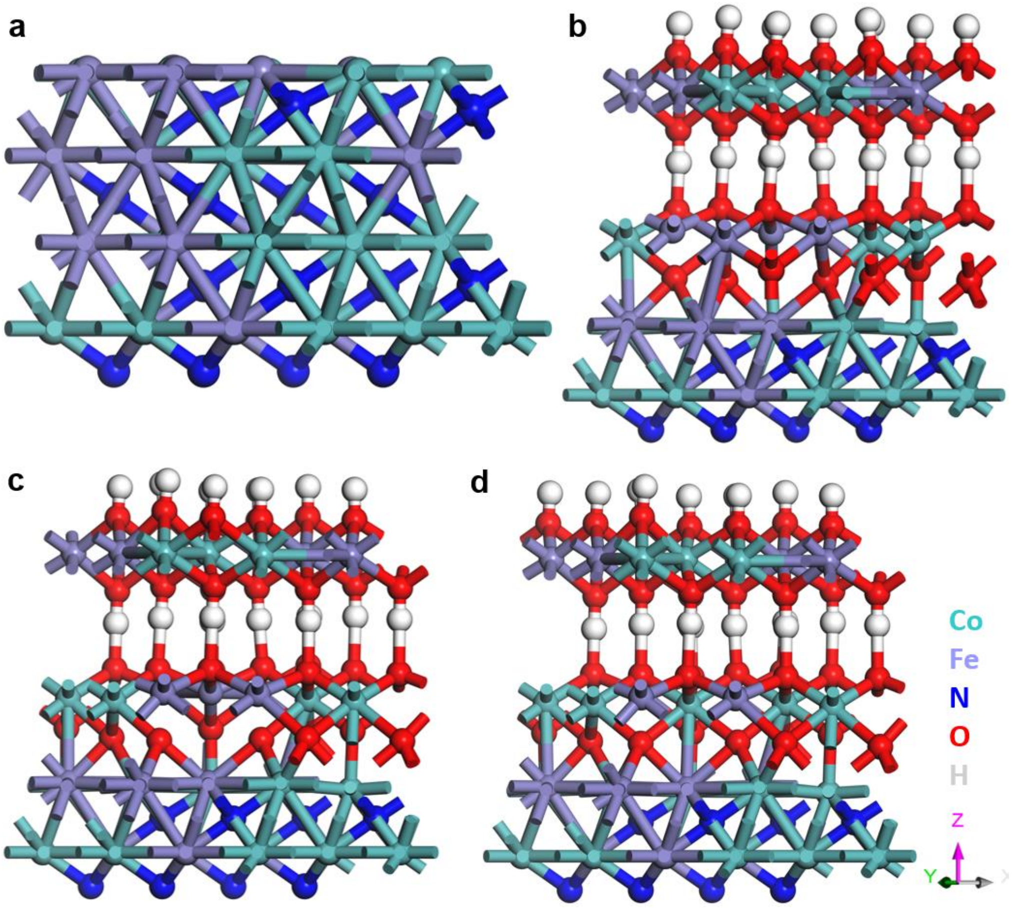

Supplementary Fig. 14 | The computational models. (**a**) (Co,Fe)$_3$N_R, (**b**) (Co,Fe)$_3$N_1D, (**c**) (Co,Fe)$_3$N_2D and (**d**) (Co,Fe)$_3$N_1D. These models were established based on the EELS results, where a new oxyhydroxide shell is identified in addition to the original nitride bulk. Therefore, two-phase models of oxyhydroxide covering nitride were selected to represent the actual configurations at the latter three stages. In oxyhydroxide layers, Co/Fe ratios were determined by EELS analyses to be 0.68 in (Co,Fe)$_3$N_1D, 0.87 in (Co,Fe)$_3$N_1C and 0.93 in (Co,Fe)$_3$N_2D.

Supplementary Table 1 | The key electrocatalytic parameters of electrocatalysts at different electrochemical stages.

Sample	$(Co,Fe)_3N$_R	$(Co,Fe)_3N$_1D	$(Co,Fe)_3N$_1C	$(Co,Fe)_3N$_2D
ORR overpotential (η_{ORR}) at –2 mA cm^{-2} (V)	0.42	0.38	0.35	0.34
ORR Tafel slope (mV dec^{-1})	57	74	69	63
OER overpotential (η_{OER}) at 10 mA cm^{-2} (V)	0.39	0.36	0.33	0.31
OER Tafel slope (mV dec^{-1})	75	86	80	77
Bifunctionality (V, η_{ORR} + η_{ORR})	0.81	0.74	0.68	0.65

Supplementary Table 2 | ELNES analyses of electrocatalysts at different electrochemical stages.

Sample		$(Co,Fe)_3N$_1D		$(Co,Fe)_3N$_1C		$(Co,Fe)_3N$_2D	
		Bulk	Shell	Bulk	Shell	Bulk	Shell
Co L-edge	L_3-edge	779.1	779.6	779.8	780.8	780.0	780.7
	L_2-edge	794.4	794.8	794.8	795.8	795.5	795.7
	L_3/L_2 Intensity ratio	4.6	4.2	4.2	3.0	4.1	3.9
	L_3 Branching Ratio	0.820	0.807	0.809	0.752	0.804	0.795
Fe L-edge	L_3-edge	708.9	708.9	708.9	709.1	709.0	709.0
	L_2-edge	721.4	721.4	721.3	721.8	722.0	722.0
	L_3/L_2 Intensity ratio	4.7	4.7	4.7	4.8	4.7	4.8
	L_3 Branching Ratio	0.824	0.826	0.826	0.829	0.825	0.827

Supplementary Table 3 | Key parameters comparison of rechargeable Zn-air batteries in recently published literatures.

Catalyst	Power density ($mW\ cm^{-2}$)	Current density ($mA\ cm^{-2}$)	Voltage gap (V)	Cycling hours (h)
$(Co,Fe)_3N$_2D	234	5	0.64	750
		30	0.85	300
Co–N_x–C[4]	78	2	1.04	79
MnO/Co/PGC[5]	172	5	~0.80	116.7
Ni, N-doped GC[6]	83.8	2	0.77	43
Co/Co-N-C[7]	132	10	0.82	333.3
3DOM-Co@Ti_xON_y[8]	110	20	0.97	300
NOGB-800[9]	111.9	10	0.72	30
Fe-SAs/NPS-HC[10]	195	5	0.96	55.6
FeCo-N_x-C[11]	150	10	0.80	40
Co–N_x-C[12]	152	2	~1.00	60
(Mg, Co)$_3O_4$@NGC[13]	125	10	0.80	200

Supplementary Note 1

To verify the proposed hypothesis of surficial evolution, the XPS spectra at surface and bulk were conducted for the air electrodes at different electrochemical states. Two major differences are demonstrated in parallel and vertical comparison. The most significant difference in the parallel comparison is the presence and absence of the nitride-feature peaks at respective bulk and surface.[14] The peaks at 779 eV for Co $2p_{3/2}$ and the ones at 398 eV for N *1s* co-existed in bulk regions but are nondetectable on the surface. The O *1s* spectra shows an inverse phenomenon with strong peak presence in shell regions while the negligible signal was shown in bulk. All indicates the different phases and compositions at the two depths. When conducting vertical comparison among spectra at the same depth, the bulk peaks shows relatively no resonance to electrochemical controls and negligible shifts are acquired for their positions. However, as for surficial elements, their peaks demonstrate visible shift along with cycling. The left shift of Co $2p_{3/2}$ as well as the satellite peak are shown in $(Co,Fe)_3N$_1C when comparing to $(Co,Fe)_3N$_1D, reflecting the increase of Co valency. Then, the three peaks shift back to their original positions for $(Co,Fe)_3N$_2D. Simultaneously, Co-O peak at about 530.7 eV in O *1s* also demonstrates a similar change upon cycling. The XPS analyses clearly confirm the hypothesis on the surficial location of electrochemically accessible Co.

Supplementary References

1 Jiang, Y. *et al.* Multidimensional ordered bifunctional air electrode enables flash reactants shuttling for high-energy flexible Zn-air batteries. *Adv. Energy Mater.* **9**, 1900911 (2019).

2 Wang, Z. L., Yin, J. S. & Jiang, Y. D. EELS analysis of cation valence states and oxygen vacancies in magnetic oxides. *Micron* **31**, 571-580 (2000).

3 Schmid, H. K. & Mader, W. Oxidation states of Mn and Fe in various compound oxide systems. *Micron* **37**, 426-432 (2006).

4 Li, B. Q. *et al.* Framework-Porphyrin-Derived Single-Atom Bifunctional Oxygen Electrocatalysts and their Applications in Zn-Air Batteries. *Adv. Mater.* **31**, 1900592 (2019).

5 Lu, X. F., Chen, Y., Wang, S., Gao, S. & Lou, X. W. D. Interfacing Manganese Oxide and Cobalt in Porous Graphitic Carbon Polyhedrons Boosts Oxygen Electrocatalysis for Zn-Air Batteries. *Adv. Mater.* **31**, 1902339 (2019).

6 Qiu, H. J. *et al.* Metal and Nonmetal Codoped 3D Nanoporous Graphene for Efficient Bifunctional Electrocatalysis and Rechargeable Zn-Air Batteries. *Adv. Mater.* **31**, 1900843 (2019).

7 Yu, P. *et al.* Co Nanoislands Rooted on Co-N-C Nanosheets as Efficient Oxygen Electrocatalyst for Zn-Air Batteries. *Adv. Mater.* **31**, 1901666 (2019).

8 Liu, G. *et al.* An oxygen-vacancy-rich semiconductor-supported bifunctional catalyst for efficient and stable zinc-air batteries. *Adv. Mater.* **31**, 1806761 (2019).

9 Hu, Q. *et al.* Trifunctional Electrocatalysis on Dual-Doped Graphene Nanorings–Integrated Boxes for Efficient Water Splitting and Zn–Air Batteries. *Adv. Energy Mater.* **9**, 1803867 (2019).

10 Chen, Y. *et al.* Enhanced oxygen reduction with single-atomic-site iron catalysts for a zinc-air battery and hydrogen-air fuel cell. *Nat. Commun.* **9**, 5422 (2018).

11 Li, S., Cheng, C., Zhao, X., Schmidt, J. & Thomas, A. Active Salt/Silica-Templated 2D Mesoporous FeCo-N_x-Carbon as Bifunctional Oxygen Electrodes for Zinc-Air Batteries. *Angew. Chem. Int. Ed.* **57**, 1856-1862 (2018).

12 Tang, C., Wang, B., Wang, H. F. & Zhang, Q. Defect engineering toward atomic Co-N_x-C in hierarchical graphene for rechargeable flexible solid Zn-air batteries. *Adv. Mater.* **29**, 1703185 (2017).

13 Deng, Y.-P. *et al.* Hierarchical porous double-shelled electrocatalyst with tailored lattice alkalinity towards bifunctional oxygen reactions for metal-air battery. *ACS Energy Lett.* **2**, 2706-2712 (2017).

14 Cruz, W. D. L., Contreras, O., Soto, G. & Perez-Tijerina, E. Cobalt nitride films produced by reactive pulsed laser deposition. *Rev. Mex. Fis.* **52**, 409-412 (2006).
